# The role of social support in the effectiveness of counseling for international students

**DOI:** 10.1080/28324765.2026.2653852

**Published:** 2026-04-07

**Authors:** Hongjun Tan, Louis G. Castonguay, Rebecca A. Janis, Jeffrey A. Hayes, Sultan A. N. Magruder, Caitlin L. Chun-Kennedy, Brett E. Scofield

**Affiliations:** a Department of Psychology, Pennsylvania State University, University Park, PA, USA; b Center for Counseling and Psychological Services, Pennsylvania State University, University Park, PA, USA; c Department of Educational Psychology, Counseling, and Special Education, Pennsylvania State University, University Park, PA, USA

**Keywords:** College counseling, international students, social support, family support, practice-oriented research

## Abstract

Previous studies on college counseling have shown that international student clients experience higher psychological distress and benefit less from individual psychotherapy in a university counseling center (UCC) setting compared to domestic student clients. International students face many challenges during adjustment to a foreign country, and social support plays a crucial role during this process and in their psychological well-being. This study aimed to compare the levels of pre-treatment symptoms and treatment outcomes between international and domestic student clients, and examine how perceived social support was related to these differences. The study used data collected from the Center for Collegiate Mental Health (CCMH), including 4876 clients treated by 1679 therapists in 98 UCCs, of which 2438 were international student clients from 142 countries. Multivariate general linear models were constructed to explore our research questions. International student clients reported lower perceived social network support and higher family support, and higher depression, academic distress and anger/frustration at pre-treatment than domestic student clients, but there were limited differences in treatment outcomes. Both perceived social network support and family support were predictive of pre-treatment symptoms, and family support was shown to be positively related to better treatment outcomes in patients with depression, anxiety and academic distress. Implications for clinical practice and future research are discussed.

## IMPACT STATEMENT

International students present to counseling with overall greater distress but achieve largely comparable treatment outcomes to domestic students. Higher perceived family support in both international and domestic student clients predicts better treatment outcomes in symptoms such as depression, anxiety, and academic distress. The results highlight the importance of assessing and integrating family support in clinical practice in university counseling centers.

International students constitute a significant proportion of the US college student population. Despite a decrease in the total number of international students during 2020–2021 due to the COVID-19 pandemic, the 2021 Open Doors® Report revealed that 914,095 international students enrolled in a US higher institution during this year, constituting 4.63% of the college student population in the United States (Institute of International Education, 2021). International students experience a wide range of unique challenges, including acculturative and financial stress, racial discrimination and xenophobia, language barriers, limited social support and the development of self-identity (Prieto-Welch, 2016; Smith & Khawaja, 2011). As a result of a combination of these factors, international students are at high risk of experiencing mental health problems. A World Health Organization study of international students across eight countries showed that almost one in three first-year international students screened positive for at least one DSM-5 disorder in the past 12 months, with ‘major depressive episode’ and ‘generalized anxiety disorder’ having the highest prevalence rates (18.5% and 16.7%, respectively; Auerbach et al., 2018).

University counseling centers (UCCs) are essential source of mental health care widely utilized by college students. Findings obtained within an extensive practice research network, the Center for Collegiate Mental Health (CCMH), which includes more than 800 UCCs across the United States, Canada and England, have demonstrated that college counseling is effective in relieving psychopathological symptoms such as academic distress, anxiety and depression, and improving academic performance and retention rates across a diverse range of college students (e.g. Lockard et al., 2019; 2013; McAleavey et al., 2019; Scofield et al., 2017). However, to date, a limited number of studies have focused on the outcomes of international student clients in counseling. The evidence gathered by these studies, however, suggested the presence of unmet mental health needs within this population.

In one study comparing the prevalence of mental health diagnoses among international and domestic students in the US, international students were less likely to report having received a formal mental health diagnosis from a professional despite reporting higher levels of depressive symptoms (Yeung et al., 2022). Consistently, previous studies also suggested that international students were less likely to seek counseling because of cognitive (e.g. mental health stigma), cultural (e.g. adherence to original cultural values) and therapist (e.g. matching of ethnicity and cultural background) factors (Kawamoto et al., 2018; Liao et al., 2005; Tedeschi & Willis, 1993). In addition, international student clients may not benefit as much from psychotherapy services provided by UCCs as domestic students. A study showed that international student clients reported higher psychological distress than domestic students after a course of treatment provided by the university counseling centers, although this gap in effectiveness was reduced when a higher proportion of international students were seen at these UCCs (Keum et al., 2022). Keum et al. (2022) suggested that therapist factors such as cultural competence and flexibility in the treatment approach, as well as the provision of interpersonal support to international student clients through outreach efforts and engaging them beyond the traditional boundaries of the therapy room, may have contributed to the differences in treatment outcomes between international and domestic student clients. These findings highlight the need for further understanding of the mechanisms that might explain this gap in treatment effectiveness between international and domestic students, and that social support outside the therapy room could potentially influence psychotherapy outcomes for international students.

Previous research identified social support as a key factor in psychological adjustment and well-being among college students. Robust empirical evidence indicates that stronger social support is predictive of better mental health outcomes (Harandi et al., 2017). Social support is particularly crucial for international students undergoing acculturation, a process of sociocultural and psychological change in their relationship with their home and host cultures as they settle into a new cultural environment (Berry, 2005). Studies have demonstrated that international students with higher levels of social support experience less acculturative stress, a factor that exacerbates psychological distress (Constantine et al., 2004; Rice et al., 2012; Wei et al., 2007; Yeh & Inose, 2003). Aside from the acculturation process, Hefner and Eisenberg (2009) reported that international students (as well as ethnic minority students and students with low socioeconomic status) were at greater risk of social isolation. Although the positive effects of social support on international students’ mental health have been well-documented, there has been limited research on whether it plays a role in the context of psychotherapy services for these students.

In recent reviews that were not specific to the college student population, social support was found to be a significant positive predictor of client improvement across various populations, diagnoses and treatment modalities (Constantino et al., 2021). Although it can be theorized that some clients' lack of social support could have reflected their existing interpersonal problems and symptomology, Zimmermann et al. (2020) demonstrated that social support as a predictor had a unique impact on treatment outcomes. One recent study specifically examined the role of social support from the local community and discovered that international student clients in treatment at UCCs who experienced lower perceived social support demonstrated higher levels of psychological distress at the end of treatment compared to their domestic counterparts, highlighting the role of perceived social support in the differential treatment outcomes between international and domestic student clients (Robbins et al., 2024).

While we know that social support is related to treatment outcomes in psychotherapy and that a gap in treatment outcomes exists between international and domestic student clients, a more comprehensive understanding of how social support relates to treatment outcomes among international student clients is critical to improving care for this population. Two prominent limitations exist within the current literature. First, studies frequently operationalize psychological distress as a univariate composite factor (e.g. Bartholomew et al., 2022; Keum et al., 2022; Robbins et al., 2024). Kawamoto et al. (2018) provided foundational insight on how international student clients' levels of distress across a broad range of psychological symptoms (e.g. depression, anxiety, academic distress, etc.) compared to domestic students while controlling for cultural backgrounds. Their study, nevertheless, mainly focused on describing psychological distress at the beginning of treatment, where a significant proportion of the client sample only attended one psychotherapy appointment instead of a course of treatment. Thus, this study aims to examine not only the levels of a range of psychological symptoms at intake, but also the trajectory of these symptoms at the end of the treatment course.

Additionally, the studies included in the review by Constantino et al. (2021) did not differentiate among sources of social support (e.g. family members, friends and professional relationships). Given the unique circumstances of international students, who typically have limited contact with family from their home country and need to build a new social network in a new country, more research is needed to further our understanding of how various sources of social support are related to the well-being and treatment effectiveness of international students. Deepening our knowledge in this topic could potentially reveal factors that could augment client improvement when clients' access to services might be limited, and draw clinicians' attention to utilizing and emphasizing these factors in their practices to enhance their effectiveness in working with this client population.

## Current study

The goal of this study was to expand on previously established knowledge about international students' mental health, social support and treatment outcomes. This study has two main aims. For Aim 1, we attempted to examine more closely the differences in psychological symptom severity between international and domestic student clients by comparing a broad range of pre-treatment symptoms and exploring how perceived social support, particularly from family and from the local personal network, was related to these symptoms. We hypothesize that international student clients would present to treatment with more severe symptomology across various domains of psychological symptoms than domestic student clients, and that both forms of perceived social support would be negatively correlated with symptom severity.

As for Aim 2, we attempted to better understand the nature of the difference in treatment outcomes between international and domestic student clients by examining their improvement in each domain of psychological symptoms relative to the beginning of treatment. Our objective was to identify specific symptoms that varied in the magnitude of symptom reduction between international and domestic student clients at the end of the treatment. In addition, we explored how family and social network support are related to treatment outcomes. Based on previous findings on this topic, we hypothesized that international student clients and clients with lower levels of perceived social support would show less symptom reduction than domestic student clients across all the measured symptom domains. For both aims, we explored the interactive effects of international student status and social support on pre-treatment and post-treatment symptoms to examine whether perceived social support moderated the potential differences in psychological distress and treatment outcomes between international and domestic student clients.

## Methods

### Participants

We utilized data from the CCMH database for the years 2017–2019 and 2021–2023. Consistent with practice-oriented research (PRN; Castonguay et al., 2021), CCMH data are collected as part of the clinical routine of its participating centers. We decided not to include the 2019–2020 and 2020–2021 academic years because of the timing of the COVID-19 pandemic, a period when institutions and students struggled to navigate through this global public health crisis. International students during this time not only faced the international travel bans that posed significant roadblocks for them to return home, but also government policies that challenged the legality of their stay in the US (see Buckner et al., 2022). These challenges were alleviated after the change in governmental administration in 2021 and the lifting of travel bans in August 2021. Thus, we included data collected in years that were not directly impacted by these issues to limit confounding effects.

Clients were deemed eligible for this study if they received at least one psychotherapy session at their UCCs and consented to contribute deidentified data to the CCMH as part of their clinical routine. We followed Hayes et al. (2016) data reduction procedures, keeping only data from the clients’ first course of treatment with at least two sessions of individual psychotherapy or clinical intake to limit the confounding effects of previous treatment experiences at the same UCC or even with the same therapist. Because our present study focused on individual treatment, clients who attended group therapy during their first course of therapy were excluded to prevent the potential confounding effects of additional services that the client received. In addition, the clients needed to have completed the SDS and at least two recorded CCAPS-34, where the first CCAPS-34 must have been recorded within 14 days of the first appointment. Post-treatment symptoms were captured by the last recorded CCAPS-34, which was completed within 3 days prior to an individual appointment; this appointment was also operationalized as the final session of this course of treatment for the clients.

The initial sample included 60,608 student clients, of whom 3462 were international student clients and 57,146 were domestic. Expanding upon the Robbins et al. (2024) study, which used all eligible clients, we used matching methods to create a more balanced dataset to address the wide gap in sample sizes between international and domestic student clients and to control for variables that may otherwise explain differences between the two groups. We determined the variables used as matching criteria from the predictors of international student mental health identified by Brunsting et al. (2018), while minimizing the amount of data pruned during the matching process. We chose the treatment center as a control variable to account for the center effect (as identified by Keum et al., 2022) as well as geographical location and local culture, which might interact with race/ethnicity to determine the students’ sense of belonging. Academic status was selected as a control for age, acculturation level, and nature of distress related to the students’ progression in their academic program. In addition to their predictive values, we chose race/ethnicity and gender as control variables to account for some of the variance in students’ experiences with discrimination.

The resulting dataset contained the same number of international and domestic student clients with the same controlled demographic covariates. The matching procedure was conducted through the MatchIt R package (Ho et al., 2011) using exact matching of international and domestic student clients based on these variables. Each international student client was randomly paired with one domestic student client of the same race, gender and academic status who was treated in the same UCC. International student clients without a matching domestic student client were not included in this study. Of the initial 3462 international student clients who met the inclusion criteria for this study, 2438 were matched and included in the final analysis. The resulting dataset contained the exact numbers of international and domestic student clients.

The analyzed sample included 4876 student clients seen by 1679 therapists in 98 UCCs. The 2438 international student clients came from 142 countries and regions. These clients attended an average of 6.49 counseling sessions (*SD* = 5.21). There were no significant differences between international and domestic student clients in the number of utilized sessions. It should be noted that although service limit policies varied across UCCs, 86% of both international and domestic student clients completed a course of treatment within 10 sessions. The average age of the participants was 23.88 years, with international student clients (*M* = 24.16) being 0.55 years older than domestic student clients (*M* = 23.61), *t*(4874) = 3.60, *p* < 0.001. A detailed breakdown of participants' demographics is presented in Table A1 of the Appendix.

### Measures

#### Standardized data set (SDS)

The SDS collects clients' basic demographic information, mental health history and academic status. In addition, the SDS contains information about the clients' therapists and treatment centers. The SDS is commonly completed digitally at intake. The primary variables for the present study collected in this measurement are international student status (Yes/No), and social support. Two items assess perceived social support from family and social networks (e.g. friends and acquaintances) separately, where clients are asked to respond to the statements ‘I get the emotional help and support I need from my family (item 1)/social network (item 2)’ on a five-point Likert-type scale ranging from 1 (strongly disagree) to 5 (strongly agree). Although these two constructs were assessed with single-item measures for actionable data collection in a large PRN context, these items have been shown to yield meaningful results in studies of social support among marginalized populations (e.g. Bartholomew et al., 2019; Robbins et al., 2024). We followed the approach of these previous studies in treating the social support variables as continuous variables. The secondary variables collected from the SDS, such as race/ethnicity, gender, etc., are used to describe the sample and used as control variables, as explained in the Statistical Analyses section.

#### Counseling center assessment of psychological symptoms-34 (CCAPS-34)

The CCAPS-34 is a 34-item multidimensional measure of a wide range of psychological distress in college students, including depression, generalized anxiety, social anxiety, academic distress, eating concerns, alcohol use and hostility (Locke et al., 2012). The subscale scores were calculated by averaging the scores of the items that loaded onto each subscale, and the possible subscale scores ranged from 0 to 4. Across the subscales, the CCAPS-34 has acceptable to good internal consistency (Cronbach's *α* ranging from 0.76 to 0.89) and one-week test-retest reliability (coefficients range = 0.79–0.87) (Locke et al., 2012). The CCAPS-34 has been shown to be invariant between international and domestic student clients, and the subscales demonstrated good internal consistency in studies including this population (Cronbach's *α* = 0.0.77–0.91; Bartholomew et al., 2023). With respect to validity, the CCAPS-34 is highly correlated with other common outcome measurements such as the Outcome Questionnaire-45 (OQ-45), the Beck Depression Inventory (BDI) and the Beck Anxiety Inventory (BAI) (Locke et al., 2012; Nordberg et al., 2018). For the present study, we used pre-treatment CCAPS-34 subscale scores to describe the clients' psychological distress, and residualized the CCAPS-34 subscale change scores to capture treatment outcomes.

### Procedure

The data utilized in this study were collected by CCMH-member counseling centers as part of their routine clinical procedures. All the clients' SDS data were collected before their first counseling appointment, and the CCAPS data were collected immediately before each of their counseling appointments. We used the first and last recorded CCAPS score for our analyses. Only the data from clients who consented to contribute data to the CCMH were de-identified and submitted to the CCMH data repository, where the data were cleaned, stored and analyzed.

### Statistical analyses

Prior to the primary analysis, we conducted a correlation analysis of family support and social network support for international and domestic student clients separately and compared whether the two types of support were similarly highly correlated between the two groups to determine whether they can be averaged into one single ‘social support’ variable or should be fitted into the model as two different variables. Our preliminary analysis showed that perceived social network support and family support were modestly correlated for both international and domestic student clients (*r* = 0.28–0.33). Thus, we fitted social network support and family support as separate constructs into our proposed models.

Our hypotheses and exploratory questions were tested using two sets of omnibus multivariate general linear models (GLM) followed by a series of follow-up univariate GLMs in which each of the seven CCAPS-34 subscales was used as the dependent variable. In the Aim 1 multivariate GLM, we included international student status, perceived family support for the full sample, perceived social network support for the full sample, and the interaction between these variables as predictors for the dependent variable. We included only statistically significant predictors of the dependent variables in the multivariate GLM as independent variables in the follow-up univariate GLM. These follow-up models explore how the independent variables relate to each of the seven domains of psychological symptoms captured by the CCAPS-34. International student status was contrast-coded, while social support variables were mean-centered before being fit into the model. Interaction terms were calculated as the product of the variables.

For Aim 2, we operationalized the treatment outcome as the residualized change scores on each CCAPS subscale. Residualized change scores estimate the difference between a client's actual outcome and the expected outcome based on prior assessments. In this analysis, we predicted post-treatment CCAPS-34 subscale scores by regressing them on the pre-treatment scores and then computed the residuals between each client's actual and predicted scores. A residualized change score of 0 indicates that the client's post-treatment symptoms match the expected level. In contrast, a positive score indicated more post-treatment symptoms than anticipated, hence a worse treatment outcome. The analytical procedure for Aim 2 was similar to that for Aim 1, while changing the dependent variable to these residualized change scores. The follow-up univariate GLMs for Aim 2 examined the effects of international student status, social support, and potentially their interaction on symptom changes in each of the seven psychological symptoms assessed by the CCAPS-34.

We reported the demographics (e.g. age, gender, ethnicity, academic status, etc.) and treatment information (e.g. number of attended sessions, attendance rate, etc.) of our sample. We included the number of attended sessions as a covariate for the Aim 2 analyses, as treatment dosage is directly related to treatment outcome (Howard et al., 1986). To account for the effect of treatment centers as documented in Keum et al. (2022) and Robbins et al. (2024), we included a fixed effect of treatment centers as a covariate in the Aim 2 models. Because a significant proportion of therapists did not see more than one client in this dataset, we did not estimate therapist effects in our analyses. Owing to the large number of models estimated in this study, we identified *p* = 0.003 (0.05/16) as the threshold for statistical significance across each step of the analyses using Bonferroni correction.

## Results

Prior to the primary analyses, we conducted a preliminary analysis to identify potential differences in perceived social support between international and domestic student clients at baseline. We found that international student clients (*M* = 3.45) reported lower perceived support from their local social network than domestic student clients (*M* = 3.68), *F*(1, 4873) = 50.99, *p* < 0.001, partial *η*^*2*^ = 0.010; however, international student clients (*M* = 3.42) reported higher perceived family support than domestic student clients (*M* = 3.26), *F*(1, 4873) = 20.63, *p* < 0.001, partial *η*^*2*^ = 0.004.

### Differences in symptoms at pre-treatment (Aim 1)

The multivariate GLM revealed significant main effects of international student status (*F*(7, 4862) = 13.07, *p* < 0.001, Pillai’s trace = 0.018), perceived family support (*F*(7, 4862) = 53.15, *p* < 0.001, Pillai’s trace = 0.071), and perceived social network support (*F*(7, 4862) = 52.44, *p* < 0.001, Pillai’s trace = 0.070) on pre-treatment psychological symptoms. The results for the follow-up univariate GLMs for each of the CCAPS-34 subscales partially supported our hypothesis. International and domestic student clients only differed on some of the subscales, where international student clients presented to treatment with higher levels of depression (*b* = 0.11, *F*(1, 4871) = 26.92, *p* < 0.001, partial *η*^*2*^ = 0.003) and anger/frustration (*b* = 0.12, *F*(1, 4871) = 33.81, *p* < 0.001, partial *η*^*2*^ = 0.006) than domestic student clients, while there were no significant differences in anxiety, social anxiety, academic distress, eating concerns and alcohol use between the two groups.

Supporting our hypothesis on the relationship between perceived family support and psychological symptoms, the follow-up analysis revealed that perceived family support across both international and domestic students was negatively correlated with all CCAPS-34 subscale scores other than alcohol use. To be exact, we found that clients with higher perceived family support experienced lower levels of depression (*b* = −0.12, *F*(1, 4871) = 302.19, *p* < 0.001, partial *η*^*2*^ = 0.020), anxiety (*b* = −0.05, *F*(1, 4871) = 55.72, *p* < 0.001, partial *η*^*2*^ = 0.004), social anxiety (*b* = −0.10, *F*(1, 4871) = 182.45, *p* < 0.001, partial *η*^*2*^ = 0.020), academic distress (*b* = −0.09, *F*(1, 4871) = 122.13, *p* < 0.001, partial *η*^*2*^ = 0.010), eating concerns (*b* = −0.04, *F*(1, 4871) = 17.08, *p* = 0.023, partial *η*^*2*^ = 0.002) and anger/frustration (*b* = −0.07, *F*(1, 4871) = 105.00, *p* < 0.001, partial *η*^*2*^ = 0.010). The relationship between perceived family support and alcohol use (*b* = −0.02, *F*(1, 4871) = 5.97, *p* = 0.015, partial *η*^*2*^ = 0.001) at pre-treatment was statistically insignificant.

Similarly, the overall client population’s perceived social network support was negatively correlated to depression (*b* = −0.22, *F*(1, 4871) = 311.03, *p* < 0.001, partial *η*^*2*^ = 0.060), anxiety (*b* = −0.11, *F*(1, 4871) = 69.18, *p* < 0.001, partial *η*^*2*^ = 0.020), social anxiety (*b* = −0.17, *F*(1, 4871) = 177.76, *p* < 0.001, partial *η*^*2*^ = 0.040), academic distress (*b* = −0.14, *F*(1, 4871) = 101.93, *p* < 0.001, partial *η*^*2*^ = 0.020) and anger/frustration (*b* = −0.08, *F*(1, 4871) = 58.09, *p* < 0.001, partial *η*^*2*^ = 0.010). However, perceived social network support was not related to eating concerns (*b* = −0.04, *F*(1, 4871) = 6.27, *p* = 0.012, partial *η*^*2*^ = 0.002) and alcohol use (*b* = 0.00, *F*(1, 4871) = 0.002, *p* = 0.965, partial *η*^*2*^ < 0.001).

**Figure 1.**
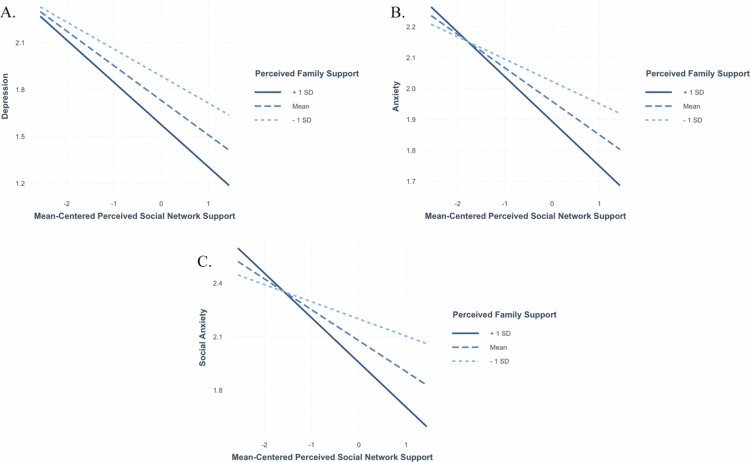
Interaction effects between perceived social network support and family support on depression, anxiety, and social anxiety. Note: Each plot represents estimates from the statistical model while holding all other variables constant at the mean.

The omnibus multivariate GLM also suggested a significant interaction effect between perceived family support and perceived social network support, *F*(7, 4862) = 8.18, *p* < 0.001, partial *η*^*2*^ = 0.004, Pillai’s trace = 0.002. Follow-up analyses showed that this interaction was significant for depression (*F*(1, 4871) = 20.10, *p* < 0.001, partial *η*^*2*^ = 0.004), anxiety (*F*(1, 4871) = 10.60, *p* = 0.001, partial *η*^*2*^ = 0.002) and social anxiety (*F*(1, 4871) = 50.10, *p* < 0.001, partial *η*^*2*^ = 0.010). The interaction effects are demonstrated in Figure 1A–C of the Appendix. In general, we found that the negative relationships between perceived social network support and these symptoms were more pronounced in clients who reported higher perceived family support. We did not find significant interactive effects between international student status and either of the perceived social support variables, nor a three-way interaction between these variables on pre-treatment symptoms.

### Differences in treatment outcomes (Aim 2)

The omnibus multivariate GLM suggested significant main effects of international student status (*F*(7, 4764) = 3.75, *p* < 0.001, Pillai's trace = 0.005) and perceived family support (*F*(7, 4764) = 3.65, *p* < 0.001, Pillai's trace = 0.005) on residualized post-treatment change in psychological symptoms. We did not detect a significant effect of perceived social network support in both international and domestic student clients on treatment outcomes, *F*(7, 4764) = 2.63, *p* = 0.010. The results for the follow-up univariate GLMs for each of the CCAPS-34 subscales, however, showed that while accounting for all other variables and covariates, international and domestic student clients only significantly differed on the improvement anger/frustration (*b* = 0.05, *F*(1,4775) = 10.75, *p* = 0.001, partial *η*^*2*^ = 0.002). On the other hand, we found that higher perceived family support across international and domestic student clients was related to more improvement on depression (*b* = −0.03, *F*(1,4775) = 16.36, *p* < 0.001, partial *η*^*2*^ = 0.005), anxiety (*b* = −0.02, *F*(1,4775) = 10.70, *p* = 0.001, partial *η*^*2*^ = 0.003) and academic distress (*b* = −0.04, *F*(1,4775) = 18.56, *p* < 0.001, partial *η*^*2*^ = 0.005) through a course of treatment across all of the clients. We did not find any interactive effect between variables in predicting treatment outcome.

## Discussion

The present study examined the role of international student status and perceived social support as predictors of baseline and post-treatment psychological distress in college counseling clients. Using a dataset that controlled for the effects of racial identity, we found that international student clients experienced more depressive symptoms, academic distress and anger/frustration than domestic student clients at intake. International student clients perceived less support from their local social network, but more family support compared to domestic student clients in a racially balanced dataset. Both family support and social network support predict the severity of psychological symptoms at intake for both international and domestic student clients. We also found a compound effect of the two types of perceived social support on pre-treatment internalizing symptoms such as depression, anxiety and social anxiety.

At the end of treatment, we discovered that international and domestic student clients differed in treatment outcome when we aggregated the symptoms measure, and that international students experienced less reduction in symptoms related to anger/frustration. For both groups, perceived family support was positively correlated with improvements in depression, anxiety and academic distress. In contrast, perceived social network support did not significantly predict symptoms at the end of treatment. However, it should be noted that despite the statistical significance criteria being met for the results we discussed, the effect sizes were minimal. Hence, the implications of these results should be considered with caution.

### Pre-treatment symptoms, international student status and social support

Our hypothesis that international students would report higher levels of pre-treatment symptoms was partially supported. Specifically, international student clients experienced higher levels of depression, academic distress and anger/frustration compared to domestic student clients, while showing similar levels of anxiety, social anxiety, eating concerns and alcohol use. Using broader categories to describe the symptoms, our results indicated that the difference in pre-treatment distress between the two groups was driven by the difference in negative emotional experiences and distress related to academic performances, rather than anxiety and externalizing behavioral issues. These results could be interpreted within the context of perceived social support, where we found that both types of social support were predictive of pre-treatment symptoms. Consistent with previous findings, we discovered that international student clients have lower perceived social network support (Robbins et al., 2024). Unlike their domestic counterparts, it is unfortunately common for international student clients who came to the United States as sojourner immigrants to experience a loss of social connections and support while adjusting to life in their host country, which exposes them to higher risks of feelings of loneliness and isolation (Sawir et al., 2007). These negative experiences were predictive of increased difficulties in cross-cultural and academic adjustment processes, and further put international student clients at higher risk of poor academic self-perception than domestic student clients (Baba & Hosoda, 2014; Yamada et al., 2014).

Additionally, we found that the two types of social support had a compounded effect on internalized symptoms such as depression, anxiety and social anxiety, where the negative relationship between perceived social network support and these symptoms became more pronounced when the clients had higher perceived family support. This interaction was not observed in externalizing symptoms in both groups. When the clients experienced good attachment with their families, a deficit in the connection with those around them might be less emotionally stressful, whereas clients who already struggled in their family connection would suffer from more internalizing symptoms when there was also a deficit in their relationships with people around them. This result also indicated that having high-quality social support from multiple sources would be an important protective factor against developing more severe internalizing symptoms for both international and domestic student clients.

However, it should be noted that international student clients reported significantly higher perceived family support than domestic student clients. This result could potentially be attributed to the emergence of communication technologies (e.g. web-conferencing, internet-based messaging, social media, etc.) over the past decade that boosted the convenience and reduced the cost and difficulty of remote communication regardless of the students being within or across country borders from their families (Bacigalupe & Bräuninger, 2017). Thus, despite being away, international student clients were able to maintain a sense of connection with their family, and this type of remote connection seemed to be experienced by international student clients as being more supportive than the connection between domestic student clients and their parents. However, further studies should explore whether the modality, frequency and consistency of these communications contributed to the difference in perceived family support between international and domestic student clients.

Overall, our findings related to pre-treatment symptoms were aligned mainly with previous findings. The consistency of these findings further emphasized the difference in baseline psychological symptoms and quality of social support between international and domestic student clients when they presented themselves to treatment, and it is crucial for practitioners to be aware of these distinctive characteristics in their work with international student clients.

### International student status and treatment outcome

Inconsistent with previous research, our hypothesis that international student clients would have higher post-treatment distress than domestic student clients was not broadly and strongly supported. Although we found a significant difference in treatment outcome between international and domestic student clients when all the outcome variables were analyzed as a set, international and domestic student clients had similar levels of improvement across most of the symptom domains. In addition, the effect size of these differences was minimal, suggesting an overall comparable treatment outcome between international and domestic student clients.

From a macro perspective, this result suggested that college counseling centers were adept at meeting the needs of international student clients. This could be a byproduct of the multicultural ethos that undergirded collegiate counseling and advancements in understanding best practices working with international student clients, which informed various aspects of clinical services, including (a) the provision of culturally responsive and adapted treatments, (b) accessible and equitable service outreach and delivery and (c) diverse educational and professional opportunities for clinical staff. On a micro level, the comparable findings between international and domestic student clients suggest a commonality in the college student experience, which can be leveraged to help normalize feelings of disappointment or shame in seeking mental health services – feelings frequently experienced among international students. It may also be that a focus on common stressors (e.g. academics, careers and relationships) provided treatment pathways that were helpful to both client populations.

One symptom domain that showed gaps in treatment effectiveness for international student clients, while controlling for other demographic variables, was anger/frustration. Drawing from models of cultural adjustment, international students may experience a heightened level of cultural shock or stress from misunderstandings, or even discrimination, from the host culture, which could be manifested in the forms of irritability, frustration and anger in addition to other negative effects (Mesidor & Sly, 2016; Trifonovitch, 1977). This specific difference in treatment effectiveness suggests the need for further development in services provided by UCCs to better account for the unique challenges of international student clients’ cultural adjustment. Moreover, symptoms related to anger/frustration have been studied much less frequently in international students’ mental health compared to other internalizing symptoms, such as depression and anxiety. Thus, efforts to examine anger/frustration in international students could offer greater insight into more effective treatment approaches for international students.

However, caution about the equivalence of treatment benefits was indicated, as Keum et al. (2022) found that international student clients reported greater psychological distress at termination than domestic student clients. The difference observed by Keum et al. was due mainly to center-level effects, where UCCs that serve a higher percentage of international students have more experience, familiarity and knowledge of best practices to treat this population. Because the focus of the current study was on client-level differences, limited insight into center-level effects was generated from our analyses. More studies are necessary to further uncover how center-level effects could contribute to various symptom reductions in international student clients.

### Social support and treatment outcome

Our hypothesis that perceived social support was negatively related to post-treatment distress was partially supported by the results, where higher perceived family support was related to overall better prognosis when predicting the treatment on all the symptoms as a set, specifically more improvement in depression, anxiety and academic distress for both international and domestic student clients; on the other hand, perceived social network support was not predictive of overall treatment outcome, while higher perceived social network support only predicted more improvement in depressive symptoms. These findings implied that the quality of perceived family support, compared to perceived social network support, could be a more useful predictor of treatment outcome, especially for patients with internalizing symptoms. We did not observe any interaction effects between social support and international student status, between the two types of social supports, nor a three-way interaction between these variables. These results suggest that each type of support functions independently in the course of the clients' treatment. We theorize that the difference in effects between the two types of social support on treatment outcomes could be partially explained by the differences in clients' service utilization. However, the effects of social support on service utilization were not the focus of this study, and more nuanced exploration and analyses are needed to better understand the relationship between social support, service utilization and treatment outcomes.

### Clinical implications

There are several important clinical implications based on the findings outlined above. The differences in the type of social support between international and domestic student clients are notable but unsurprising. Clinicians are reminded that family support has varying levels of importance to international student clients and can have a profound impact on psychological adjustment. As such, assessing the outset of treatment, the quality of family relationships and the viability of engaging in family support throughout the course of treatment seems worthwhile. It is also vital to consider that family support may look very different based on cultural norms and expectations, likely affecting how international student clients respond to family engagement. For example, an international student client's perception of family support is conditional and based upon the client choosing a specific major, following an approved career path, and demonstrating filial piety or unconditional love based solely on the client choosing what seems best for their own life?

Importantly, international students often encounter difficulties in providing meaningful social support when acclimating to the collegiate environment, which can engender experiences of loneliness and social isolation, contributing to adverse psychological outcomes. This reality was amplified during the peak of the COVID-19 pandemic, when many international students experienced heightened levels of isolation, extended periods of separation from family in their home country, and difficulty connecting with others and receiving support during pandemic lockdowns and travel restrictions. Many international students also experienced xenophobia and racism, which may have had a negative effect on their willingness to seek personal and professional support at their universities. Treatment can tend to address the barriers that inhibit social support and help to open these pathways to connection, including addressing fears or concerns or practicing social skills. For some international student clients, it may be beneficial to augment individual therapy with other services, such as therapy groups exclusively for, or centering the experiences of international students, and campus support groups for international college students.

Despite the similarities that may exist among international and domestic student clients, clinicians are reminded to remain vigilant against imparting a *universal* treatment approach to the care of international student clients. International students are composed of individuals from various parts of the world with distinct cultures and histories. These cultural variations inform the prevalence of mental health distress, susceptibility to different stressors, expression of distress, help-seeking, and treatment response among international student clients. Therefore, it is encouraged that clinicians adopt a holistic framework when working with international student clients that incorporates an *intersectional* lens, including an understanding of the sociopolitical forces impacting international student clients.

### Implications for college and university administration

As colleges and universities in the United States continue to attract international students, they are encouraged to invest in a comprehensive range of services to support the myriad psychological, social, academic, financial and spiritual needs of these students. Fortunately, the findings of this study suggest that college and university counseling centers can effectively support the mental health needs of international student clients, though *upstream* prevention might be beneficial for averting or mitigating stressors that may lead to more severe mental health outcomes.

First, colleges and universities are likely to benefit from developing and maintaining robust orientation programs comprising information and workshops detailing the academic environment and elements that promote well-being and student success, particularly for international students. Second, it would seem that student affairs and academic units should seek to collaborate by offering culturally informed and affirmative programming that would instill stronger social support. Promoting and supporting inclusive and accessible social opportunities may help students to form social bonds that can provide practical benefits and promote resiliency in response to acculturative stress, potentially reducing the need for *downstream* interventions (e.g. mental health treatment). Furthermore, universities should consider strategies to encourage family engagement through students' academic careers, especially for the families of international students, to capitalize on the positive effects of family connection on students' mental health. Finally, it may be beneficial for college and university communities to engage in campus-wide initiatives to mobilize various stakeholders (e.g. students, staff, faculty and administrators) to offer more robust support for the psychological well-being of international students. Studies have shown that students are likely to utilize family, friends and professors for psychological support before turning to professional services (Bradley et al., 1995; Brunsting et al., 2021; Hwang et al., 2014; Lian et al., 2020). Thus, it seems incumbent to equip all staff, faculty members and students with the skills to identify, support and refer when encountering international students in psychological distress (e.g. Red Folder Initiative). Collegiate settings boast various types of peer support services (e.g. peer health educators), which can be utilized or adapted to meet the needs of international students.

### Limitations

This study also has several limitations. Firstly, it only examined the two time points at pre- and post-treatment, which does not fully reveal the more detailed process across the course of treatment. Perceived social support was only measured at pre-treatment with one singular item for each source of support, so we are not able to quantify whether there are changes in these areas throughout treatment, nor use a more nuanced perspective in understanding these constructs in this study. Similarly, although we treated the two social support items as continuous variables, Likert-scale items are ordinal by nature. Thus, using more comprehensive measurements of social support may better reflect the wide range of sources of social support experienced by the clients as well as enhancing the quality of statistical inference. Additionally, in this study, there is a small proportion of clients who continued to receive treatment after the last recorded outcome measurement; thus, their recorded number of utilized sessions did not perfectly match the actual number of utilized sessions. Moreover, it should be acknowledged that therapists could vary in their level of competence in working with international student clients, which could uniquely contribute to the observed gaps in treatment effectiveness between international and domestic student clients. Therapist effects were not examined in this study because most therapists only had one client present in the matched dataset, which would vastly influence the accuracy of the estimation of therapist effects. Nevertheless, it would be beneficial for future studies to focus on therapist factors (e.g. case-mix, cultural competence, etc.) in understanding treatment effectiveness for international students. Finally, we should acknowledge that the international student population is not a monolith. The experiences of international students may vary depending on how similar their home culture is to that of their host country. Furthermore, exposure to psychotherapy services and mental health literacy could differ widely across countries where students come from. Thus, future studies need to consider more nuanced characteristics of international students (e.g. country/region of origin, acculturation level, native language, etc.) to better understand the mental health and treatment for this population.

## Conclusion

This study aimed to examine the relationships between international student status, perceived social support and pre-treatment symptoms and treatment outcomes in UCC clients. The results of pre-treatment suggested that (1) international student clients reported lower social network support than domestic student clients, but higher family support; (2) international student clients presented higher depression, academic distress, and anger/frustration than domestic student clients at intake; (3) clients who reported higher perceived social support presented fewer psychological symptoms at intake; and (4) the effects of perceived social network support and family support depended on each other in predicting depression, anxiety and social anxiety at intake, where the negative relationship between perceived social network support and these symptoms was more noticeable when the client perceived higher family support. For treatment outcomes, we found that international and domestic student clients were similar in their improvement of their symptoms through a course of treatment, while international student clients reported higher anger/frustration at the end of treatment. Clients reporting higher perceived family support showed more improvement in depression, anxiety and academic distress. Future studies should expand upon these findings to further understand the impact of international student status and social support on the process and outcome of psychotherapy. At the same time, interventions could integrate social support as a treatment factor to improve outcomes when working with international student clients.

## Data Availability

All de-identified data, analysis code and study materials are stored within the CCMH repository, which can be made available upon request by email (ccmh@psu.edu). This study's design and its analyses were not pre-registered.

## List of abbreviation

CCMH: Center for Collegiate Mental Health
UCC: University counseling center
US: the United States of America
COVID-19: Coronavirus disease 2019
SDS: Standardized data set
CCAPS-34: Counseling center assessment of psychological symptoms-34
GLM: General linear model

## Appendix

**Table A1.** Participants’ demographics in the matched dataset.

	Total (*N* = 4876)		ISC (*N* = 2438)		DSC (*N* = 2438)	
Variable	*n*/m*ean*(*SD*)	%	*n*/m*ean*(*SD*)	%	*n*/m*ean*(*SD*)	%
**Age**	23.88 (5.37)		24.16 (5.50)		23.61 (5.22)	
**Gender**						
Woman	3134	64.27%	1567	64.27%	1567	64.27%
Man	1716	35.19%	858	35.19%	858	35.19%
Transgender man & woman	2	0.04%	1	0.04%	1	0.04%
Non-binary	12	0.25%	6	0.25%	6	0.25%
Self-identified	12	0.25%	6	0.25%	6	0.25%
**Race/ethnicity**						
African American	436	8.94%	218	8.94%	218	8.94%
American Indian/Alaskan	2	0.04%	1	0.04%	1	0.04%
Asian American/Asian	2020	41.43%	1010	41.43%	1010	41.43%
Hispanic/Latino/a	716	14.68%	358	14.68%	358	14.68%
Native Hawaiian/Pacific Islander	2	0.04%	1	0.04%	1	0.04%
White	1264	25.92%	632	25.92%	632	25.92%
Multiracial	166	3.40%	83	3.40%	83	3.40%
Other/self-identified	270	5.54%	135	5.54%	135	5.54%
**Academic status**						
Undergraduate	2964	60.79%	1482	60.79%	1482	60.79%
Graduate and professional degree student	1912	39.21%	956	39.21%	956	39.21%
**Treatment factors**						
Average number of attended sessions	6.49 (5.21)		6.59 (5.36)		6.39 (5.06)	
Number of therapists	1679		1161		1223	
Number of UCCs	98		98		98	
